# Supplementary figures and images for: Sex-specific pubertal and metabolic regulation of Kiss1 neurons via Nhlh2
Source: eLife. 2021 Sep 8;10:e69765. doi: 10.7554/eLife.69765 (PMC8439651; doi:10.7554/eLife.69765)

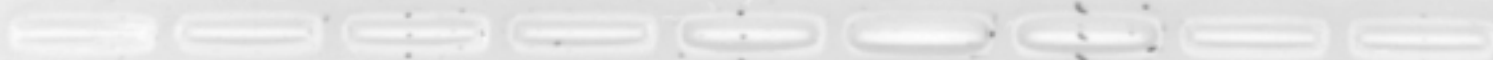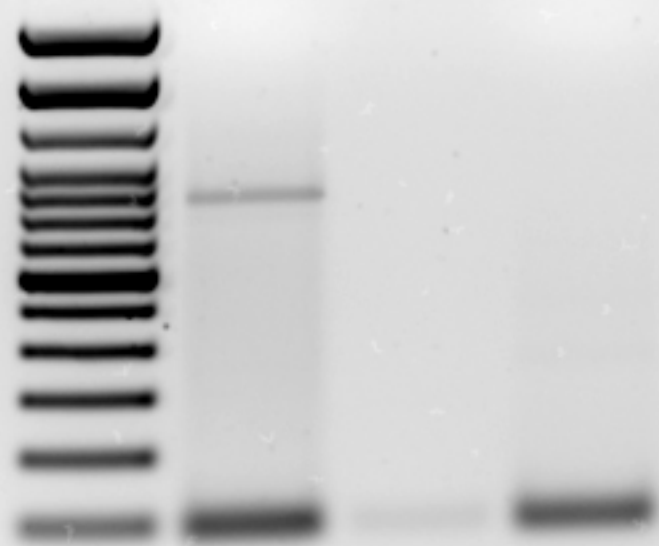

Supplement: Figure 2—source data 1. [file elife-69765-fig2-data1.pdf]

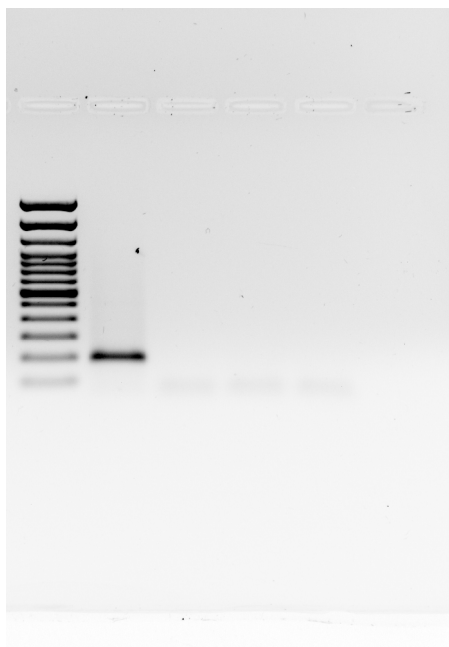

Supplement: Figure 2—source data 2. [file elife-69765-fig2-data2.pdf]

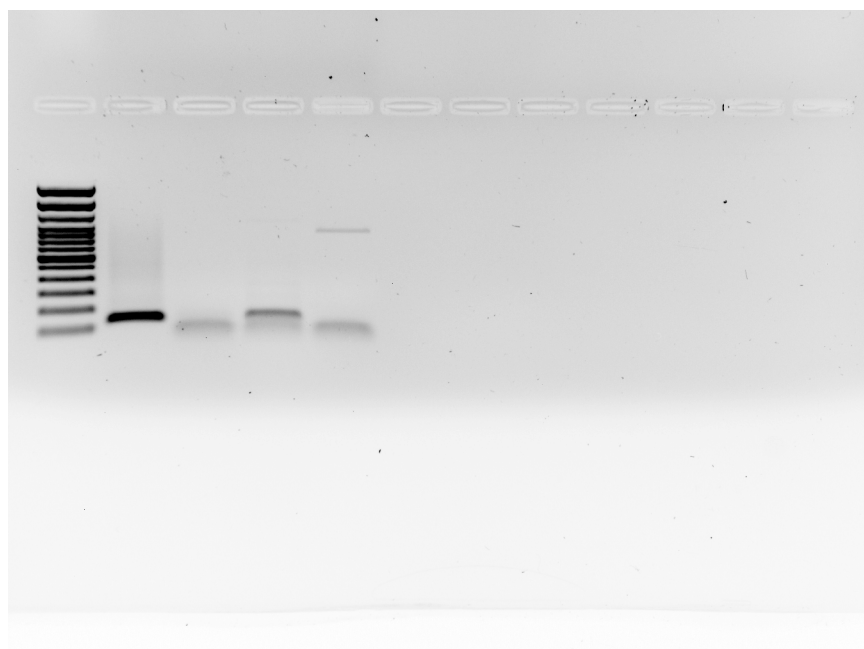

Supplement: Figure 2—source data 3. [file elife-69765-fig2-data3.pdf]

## Slide 1
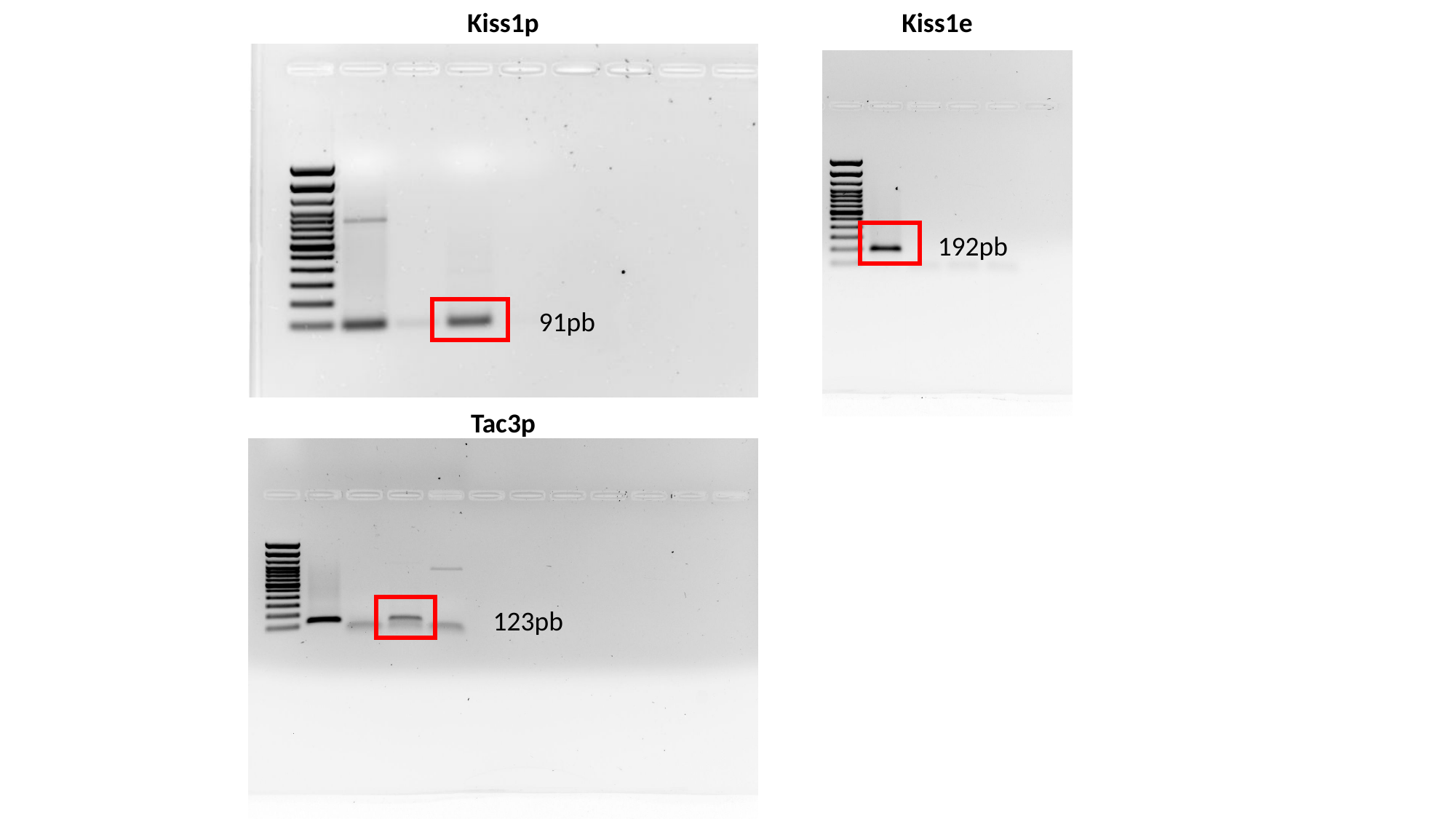

Kiss1p
91pb
Kiss1e
192pb
Tac3p
123pb

Supplement: Figure 2—source data 4. [file elife-69765-fig2-data4.pptx]
