# Supplementary material for: Sex-specific pubertal and metabolic regulation of Kiss1 neurons via Nhlh2
Source: eLife. 2021 Sep 8;10:e69765. doi: 10.7554/eLife.69765 (PMC8439651; doi:10.7554/eLife.69765)
Supplement: Transparent reporting form [file elife-69765-transrepform.docx]

***eLife’s* transparent reporting form**

We encourage authors to provide detailed information *within their submission* to facilitate the interpretation and replication of experiments. Authors can upload supporting documentation to indicate the use of appropriate reporting guidelines for health-related research (see [EQUATOR Network](http://www.equator-network.org/%20)), life science research (see the [BioSharing Information Resource](https://biosharing.org/)), or the [ARRIVE guidelines](http://www.plosbiology.org/article/info:doi/10.1371/journal.pbio.1000412) for reporting work involving animal research. Where applicable, authors should refer to any relevant reporting standards documents in this form.

If you have any questions, please consult our Journal Policies and/or contact us: [editorial@elifesciences.org](mailto:editorial@elifesciences.org).

**Sample-size estimation**

- You should state whether an appropriate sample size was computed when the study was being designed
- You should state the statistical method of sample size computation and any required assumptions
- If no explicit power analysis was used, you should describe how you decided what sample (replicate) size (number) to use

Please outline where this information can be found within the submission (e.g., sections or figure legends), or explain why this information doesn’t apply to your submission:

Power calculation was performed using Wilcoxon rank-sum method assuming non-normal distribution of the outcome with the help of the Harvard Catalyst Biostatistics Core. A sample size of 10 mice in each group will allow us to detect a difference between two groups with a two-sided alpha of 0.05 and a power of at least 84%. The calculation was performed using nQuery Adviser® v 7.0. In experiments where two groups are compared, Student’s *t*-test were performed. For comparisons of more than two groups, the statistical level were determined using ANOVA followed by the corresponding *post hoc* test depending on what kind of ANOVA (one-way, two-way, repeated measure) was used. Specific information on size and statistical analysis can be found in the material and methods and in the figure legends within the mansucript.

**Replicates**

- You should report how often each experiment was performed
- You should include a definition of biological versus technical replication
- The data obtained should be provided and sufficient information should be provided to indicate the number of independent biological and/or technical replicates
- If you encountered any outliers, you should describe how these were handled
- Criteria for exclusion/inclusion of data should be clearly stated
- High-throughput sequence data should be uploaded before submission, with a private link for reviewers provided (these are available from both GEO and ArrayExpress)

Please outline where this information can be found within the submission (e.g., sections or figure legends), or explain why this information doesn’t apply to your submission:

We were blinded to whether our data are derived from our experimental or control groups in our data analysis to avoid experimental bias, and we are rigorous and objective in our interpretation and reporting of our results. We use sufficient numbers of mice in our studies as guided by our power calculations and perform rigorous statistical analyses. All individual values are presented in the figures and no outliers were excluded. For gels, they were cropped to fit the figure but not altered in any way that compromise the results (see raw images for confirmation). All the information required for the interpretation of the data is included in the methods and figure legends.

**Statistical reporting**

- Statistical analysis methods should be described and justified
- Raw data should be presented in figures whenever informative to do so (typically when N per group is less than 10)
- For each experiment, you should identify the statistical tests used, exact values of N, definitions of center, methods of multiple test correction, and dispersion and precision measures (e.g., mean, median, SD, SEM, confidence intervals; and, for the major substantive results, a measure of effect size (e.g., Pearson's r, Cohen's d)
- Report exact p-values wherever possible alongside the summary statistics and 95% confidence intervals. These should be reported for all key questions and not only when the p-value is less than 0.05.

Please outline where this information can be found within the submission (e.g., sections or figure legends), or explain why this information doesn’t apply to your submission:

This information can be found in the figure legends.

(For large datasets, or papers with a very large number of statistical tests, you may upload a single table file with tests, Ns, etc., with reference to sections in the manuscript.)

**Group allocation**

- Indicate how samples were allocated into experimental groups (in the case of clinical studies, please specify allocation to treatment method); if randomization was used, please also state if restricted randomization was applied
- Indicate if masking was used during group allocation, data collection and/or data analysis

Please outline where this information can be found within the submission (e.g., sections or figure legends), or explain why this information doesn’t apply to your submission:

All mice with the same genotype where randomized to receive either the control or the experimental treatment. Data collection for phenotypic studies was performed blinded to genotype.

**Additional data files (“source data”)**

- We encourage you to upload relevant additional data files, such as numerical data that are represented as a graph in a figure, or as a summary table
- Where provided, these should be in the most useful format, and they can be uploaded as “Source data” files linked to a main figure or table
- Include model definition files including the full list of parameters used
- Include code used for data analysis (e.g., R, MatLab)
- Avoid stating that data files are “available upon request”

Please indicate the figures or tables for which source data files have been provided:

Uncropped gels of the ChIP assay are included in the submission.
